# Factors Associated with Increased Mortality in a Predominantly HIV-Infected Population with Stevens Johnson Syndrome and Toxic Epidermal Necrolysis

**DOI:** 10.1371/journal.pone.0093543

**Published:** 2014-04-02

**Authors:** Lauren Knight, Rudzani Muloiwa, Sipho Dlamini, Rannakoe J. Lehloenya

**Affiliations:** 1 Medical Intern, Groote Schuur Hospital, Cape Town, South Africa; 2 Department of Paediatrics and Child Health, University of Cape Town, Cape Town, South Africa; 3 Division of Infectious Diseases and HIV Medicine, Department of Medicine, University of Cape Town, Cape Town, South Africa; 4 Division of Dermatology, Department of Medicine, University of Cape Town, Cape Town, South Africa; National Institute of Infectious Diseases, Japan

## Abstract

**Introduction:**

Stevens-Johnson syndrome (SJS) and toxic epidermal necrolysis (TEN) are life-threatening drug reactions with a higher incidence in HIV-infected persons. SJS/TEN are associated with skin and mucosal failure, predisposing to systemic bacterial infection (BSI), a major cause of death. There are limited data on risk factors associated with BSI and and mortality in HIV-infected people with SJS/TEN.

**Methods:**

We conducted a retrospective study of patients admitted to a university hospital with SJS/TEN over a 3 year period. We evaluated their underlying illnesses, eliciting drugs, predictive value of bacterial skin cultures and other factors associated with mortality and BSI in a predominantly HIV-infected population by comparing characteristics of the patients who demised and those who survived.

**Results:**

We admitted 86 cases during the study period and 67/86(78%) were HIV-infected. Tuberculosis was the commonest co-morbidity, diagnosed in 12/86(14%) cases. Skin cultures correlated with BSI by the same organism in 7/64(11%) cases and 6/7 were Gram-negative. Two of the 8 cases of Gram-negative BSI had an associated Gram-negative skin culture, although not always the same organism. All 8 fatalities had >30% epidermal detachment, 7 were HIV-infected, 6 died of BSI and 6 had tuberculosis.

**Conclusions:**

Having >30% epidermal detachment in SJS/TEN carries an increased risk of BSI and mortality. Tuberculosis and BSI are associated with higher risk of death in SJS/TEN. Our data suggests there may be an association between Gram-negative BSI and Gram-negative skin infection.

## Introduction

Stevens-Johnson syndrome (SJS) and toxic epidermal necrolysis (TEN), also collectively known as Lyell's syndrome, are rare life-threatening cutaneous drug reactions with a reported combined incidence of approximately 0.05–3 cases/million per year, although considerably higher in HIV-infected persons [1], [2]. SJS and TEN are considered as a spectrum of the same disease. In SJS there is <10% of epidermal detachment and in TEN there is >30%. SJS /TEN overlap lies between these two extremes [3]. The disease manifests clinically as epidermal stripping and baring of large areas of the dermis and mucosal membranes with resultant skin and mucosal failure. The skin failure is associated with loss of haemostatic function, fluid and electrolyte imbalance, metabolic abnormalities, impaired thermoregulation and loss barrier function allowing potential pathogens to gain access to the body predisposing to localised and bacterial systemic infection (BSI) [4].

In SJS/TEN BSI is the leading cause of mortality, which can be up to 40% in tertiary centres and intensive care units with optimal care and facilities [5], [6]. The time to re-epithelialization depends on the extent of epidermal stripping which can take a few weeks. During this time the patient is susceptible to bacterial infections, both cutaneous and systemic [1], [7]. Mucosal erosions, a hallmark of SJS/TEN, provide a further portal of entry for pathogens. A retrospective study in France by de Prost and colleagues showed that *Staphylococcus aureus*, *Pseudomonas aeruginosa* and *Enterobacteriaceae* were the most common organisms cultured in the blood of patients with SJS/TEN, implicating both the skin and the gastrointestinal tract as possible portals of entry by pathogens [8].

To date there is no definitive data guiding use of prophylactic antibiotics in SJS/TEN, consensus being restricting their use to culture proven infections or the presence of clinical signs of infection [9]. de Prost and colleagues also investigated the predictive value in BSI of routine skin surface cultures. They found that skin cultures displayed excellent negative predictive value for bloodstream infections due to methicillin resistant *Staphylococcus aureus* and *Pseudomonas aeruginosa* but not for those due to Enterobacteriaceae. It is important to note that the positive predictive values were poor for all pathogens in this series [8]. The study was conducted in a developed country with a predominantly HIV-uninfected population. In our setting SJS/TEN is predominantly associated with HIV infection and it is not clear whether the risks associated with BSI are similar in HIV-infected and HIV-uninfected populations. Therefore we conducted a retrospective study of patients admitted to a university hospital with SJS/TEN. Our aim was to describe the epidemiology of BSI and the utility of skin surface cultures to predict BSI in a limited resource setting with a predominantly HIV-infected population.

## Methods

### Study setting

The study population comprised of patients presenting with SJS/TEN to a dermatology ward at Groote Schuur Hospital, a tertiary referral centre in Cape Town, South Africa. The hospital is one of two serving the population of Cape Town (approximately 3.7 million) through primary health care clinics, district and secondary hospitals [10]. The study was approved by the Human Research Ethics Committee of the University of Cape Town. As the study is a retrospective clinical record review, the approval was granted without the need for an informed consent, provided there were no identifiers of any of the participants.

### Participants and data extraction

We reviewed clinical records of consecutive patients admitted with SJS, TEN and SJS/TEN overlap to the dermatology ward from 1^st^ January 2009 to 31^st^ December 2011. Patients were admitted to a general dermatology ward managed by experienced nursing staff and doctors while admission to the intensive care unit (ICU) was only in cases of organ (other than skin) failure meeting the hospital's ICU admission policy. Patient management followed our standard protocol which is mainly supportive including fluid resuscitation, enteral nutritional support, daily baths with antiseptic solution and sterile non-adherent dressings. No physical debridement was performed, indwelling catheters were not routinely used and intravenous lines were only used when intravenous antibiotics or urgent resuscitation were necessary. No prophylactic antibiotics, systemic steroids, intravenous immunoglobulin, cyclosporine or other specific therapeutic medications were administered to any of the patients. Anticoagulation was only administered to patients who were completely bed-bound for more than 7 days.

The following data parameters were extracted from the clinical records: age; sex; HIV status; CD4 count; co-morbidities; medication used in the preceding 8 weeks; interval between the first symptoms of SJS/TEN and admission to our centre; classification as SJS, SJS/TEN overlap and TEN; vital signs; clinical management before and during admission to hospital; results of bacterial cultures; length of stay in hospital and the final outcome.

### Sampling and microbiologic definitions

Samples for bacterial skin or blood cultures were performed at the discretion of the treating physician. Skin swabs were obtained from areas that looked infected clinically. Blood samples for culture were collected from peripheral veins on clinical suspicion of BSI. Diagnosis of skin sepsis and bloodstream infection was based on the Centres for Disease Control and Prevention (CDC)/National Healthcare Safety Network (NHSN) definition of healthcare-associated infections [11].

### Statistical analysis and data presentation

Data was analysed using *Stata Statistical Software* (*Release 13*. College Station, TX: StataCorp LP). Categorical variables were summarized as percentages while continuous variables were reported as medians with interquartile range (IQR). Fisher's exact test was used to compare association between categorical variables. Two-tailed p values <0.05 were considered statistically significant.

## Results

**Figure 1:**
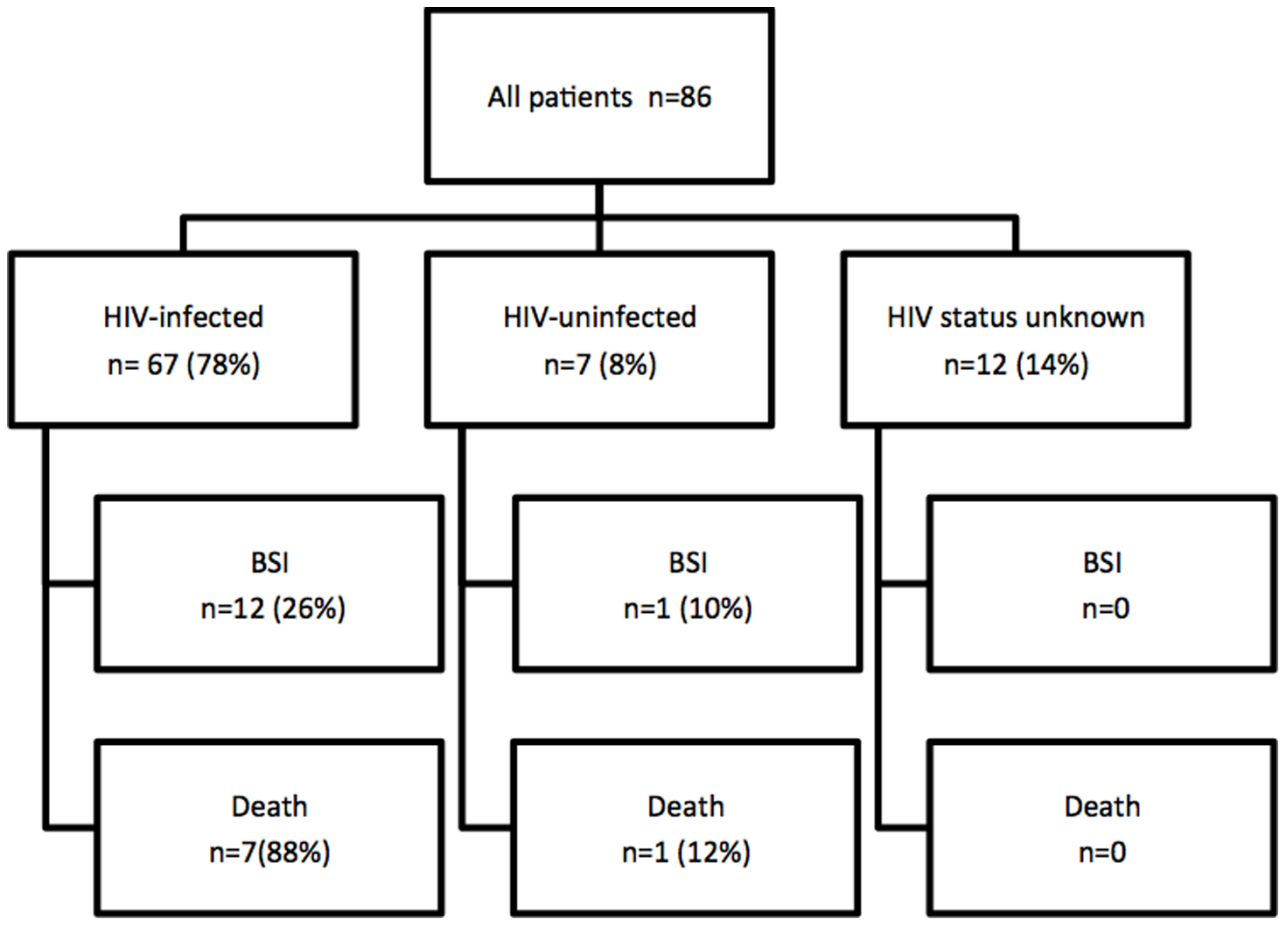
The study population according to HIV status and the outcome variables of BSI and mortality.

We admitted 86 patients with SJS/TEN to the dermatology ward during the study period. Sixty-eight (79%) were female and 18 (21%) were male with a combined median age of 32 years (IQR 27–38). Forty-five (52%) of the patients had SJS, 25(29%) had TEN and the remaining 16 (19%) had SJS/TEN overlap. Sixty-seven of the 86 (66%) cases were HIV-infected, with the median CD4 count amongst those being 137cells/mm^3^ (IQR 114 &291). Of these 68 only 28% were on antiretroviral therapy (ART). **Figure 1**. Thirty-eight of the 86 patients (44%) had a co-morbidity on admission, the commonest being tuberculosis diagnosed in 12/86 (14%) cases. Two of the 12 cases had disseminated TB, 1 other case was of drug-resistant TB with this patient being on MDR-TB treatment. All patients with TB were HIV-infected.

Nevirapine was suspected to be the offending drug in 43/86 (50%) of the cases followed by co-trimoxazole in 31/86 (36%), antiepileptics in 13/86 (15%) and anti-tuberculosis agents in 3/86 (3%). The median delay from the onset of symptoms to tertiary hospital admission was 5 days (IQR 3–7). The median time interval between onset of symptoms of SJS/TEN and clinical signs of BSI was 14 days (range 3–45days). No cases in the study were admitted to ICU as per our hospital's ICU admissions criteria.

Skin swabs for bacterial culture were performed in 35/86 patients, with a median of 3 (range 1–28) swabs per patient and 77% of these cultured an organism. For analysis, we considered multiple positive cultures of the same organism as a single positive culture, bringing the total number of positive skin swabs to 64. Blood cultures were performed on 62/86 (72%) and 32/62 (52%) had more than 1 culture done for a total of 146 cultures (range1–14). Sixteen percent grew a pathogenic organism. If an organism was cultured more than once from the same patient at different times, for our analysis we considered all of them as a single positive blood culture for that organism. Based on this there were 20 positive bacterial cultures in 13 patients. The most commonly cultured organisms were: *Acinetobacter baumannii* in 3/20, methicillin-resistant *Staphylococcus aureus* in 3/20 and *Enterobacter faecalis in* 2/20.

Four out of the 8 cases of Gram-negative BSI had an associated Gram-negative skin culture. Positive skin cultures correlated with BSI caused by the same organism in only 5/64 (8%) cases. Four of the five cases of a positive correlation between skin and blood culture grew a Gram-negative bacteria. However, the skin cultures predicted the bacterial species causing fatal BSI in only 2/7 fatal cases due to BSI. The baseline characteristics, organisms cultured are summarised in Table 1.

**Table 1:** Characteristics and culture results of patients who had BSI.

Patient	HIV status	Severity	Days to admission	Co-morbidities	Skin culture	Duration between positive skin culture & bacteraemia (days)	Blood culture	Days admitted	Outcome
1	Negative	TEN	8	Hypertension Hepatitis & Epilepsy	Not done	N/A	*S. epidermidis*	12	Discharged
2	Positive	SJS	6	None	Not done	N/A	Methicillin-susceptible *S. aureus*	9	Discharged
3	Positive	TEN	3	None	Negative culture	N/A	MRSA	37	Discharged
4	Positive	SJS	4	Pneumonia	Not done	N/A	Coagulase negative staphylococcus	9	Discharged
5	Positive	TEN	5	Epilepsy	*E. faecalis*	1	*E. faecalis*	18	Discharged
6	Positive	TEN	2	None	*K. pneumoniae & A. baumannii*	2	*K. pneumoniae*	36	Discharged
7	Positive	SJS/TEN	3	None	*MSSA*	0	MSSA & coagulase negative staphylococcus	12	Discharged
8	Positive	TEN	2	None	*E.cloacae & A. baumannii,*	6	*E. cloacae& A. baumannii*	13	Demised
9	Positive	TEN	5	Toxoplasmosis	Not done	N/A	MRSA	9	Demised
10	Positive	TEN	17	TB	*A. baumannii*	2	*A. baumannii*	17	Demised
11	Positive	TEN	5	TB	Not done	N/A	*A. baumannii*	7	Demised
12	Positive	TEN	8	TB	Not done	N/A	*E. faecium & E. cloacae*	3	Demised
13	Positive	TEN	20	TB	Not done	N/A	MRSA, *P. mirabilis*	43	Demised

*Abbreviations: A. baumannii =  Acinetobacter baumannii, E. cloacae =  Enterobacter cloacae, E. faecalis =  Enterococcus faecalis, K. pneumoniae =  Klebsiella pneumoniae, MRSA = Methicillin-resistant Staphylococcus aureus, MSSA = Methicillin-susceptible Staphylococcus aureus, P. mirabilis = Proteus mirabilis, S. aureus = Staphylococcus aureus, S. epidermidis = Staphylococcus epidermidis,*

SJS =  Stevens Johnson syndrome; TEN =  toxic epidermal necrolysis; TB =  tuberculosis.

Eight participants died during the study period for an overall mortality of 9%. All 8 had TEN and 6 died as a result of BSI, one fatality was due to hypovolemic shock and one was presumed to be due to sepsis based on clinical indices despite a negative blood culture. The 5/6(83%) fatal cases with BSI were due to a Gram negative organism, 2/5(40%) of the cases with Gram negative had a Gram negative cultured from the skin. No case of Gram negative BSI had a negative Gram negative skin culture. Seven of the 8 fatalities were HIV-infected with a median CD4 count of 87 cells/mm^3^ and 5 of the 7 were co-infected with tuberculosis. Table 2 outlines characteristics of participants that died as compared to those who survived.

**Table 2:** Variables associated with mortality.

	Died (n = 8)	Alive (n = 78)	P value#
	n(%)	n(%)	
Tuberculosis n = 86	6(63)	7(9)	<0.01
Positive HIV n = 79	7(88)	60(85)	1
BSA >30% n = 86	8(100)	33(42)	<0.01
Age >40 years n = 86	2(25)	17(22)	1
BSI (Any) = 62	6(75)	7(13)	<0.01
BSI (Gram+) n = 62	2(25)	6(11)	0.27
BSI (Gram−) n = 62	5(63)	2(4)	<0.001

# Fisher's exact test.

BSA - affected Body surface area %; BSI - Blood stream infection.

## Discussion

Our main findings in this retrospective study of 86 predominantly HIV-infected cases of SJS/TEN are the following: 1) Extent of epidermal detachment correlates with an increased risk of BSI as well as mortality. 2) BSI is strongly associated with death in SJS/TEN. 3) Co-infection with pulmonary tuberculosis increases the risk of death in HIV-associated SJS/TEN 10-fold. 4) There seems to be an association between Gram negative BSI and Gram negative skin infection although the population size in this study was inadequate to confirm this finding.

The association of tuberculosis and mortality is significant in the setting of high HIV prevalence, 78% of the study population in this series. Tuberculosis and other opportunistic infections is often the presenting feature of advanced immunosuppression in our population, hence only 24% of patients in this study were on antiretroviral therapy. Our standard of care was to treat the underlying opportunistic infection to reduce the antigen burden and minimise the risk of immune reconstitution inflammatory syndrome. Due to the retrospective nature of the study we were unable to exclude the presence of additional factors known to be risk factors for mortality in patients with HIV and tuberculosis. Five of the eight (63%) fatal cases were infected with tuberculosis, while active tuberculosis was confirmed in only 14% of the study population. The cases with tuberculosis and HIV co-infection had a comparatively lower baseline CD4 count with a median count of 87 cells/mm^3^ compared to 220 cells/mm^3^ for the study population suggesting that the HIV was at an advanced stage in those with tuberculosis. This is in comparison to the other co-morbidities in our series, which were predominantly chronic conditions that were less likely to influence mortality in acute settings. Our findings should alert clinicians of poorer outcomes when SJS/TEN occurs in patients with tuberculosis. However larger studies are needed to corroborate our findings and determine appropriate interventions to reduce mortality in patients with tuberculosis who develop SJS/TEN.

Our findings that Gram-negative bacteremia was significantly associated with mortality should influence the empirical choice of antibiotics in suspected cases of BSI following SJS/TEN. The source of these organisms has not been firmly established. The possibilities are: 1) Hospital acquired infection particularly for *A. baumanii* [12]; 2) Extensive erosion of mucosal surfaces of the gastrointestinal tract in SJS/TEN allowing for Gram negative organisms colonizing these areas easier passage into the blood stream [13], [14]; 3) Contamination of peri-anal skin with faecal matter in bed-bound patients with resultant seeding into the bloodstream. Further prospective studies are needed to determine the source of organisms infecting the skin and blood in SJS/TEN as well the extent of mucosal involvement in SJS/TEN and its correlation with the risk of BSI.

We confirmed previous findings by de Prost and colleagues that the extent of total epidermal detachment is associated with both higher incidence of BSI and mortality [8]. TEN, which by definition involves more than 30% body surface area (BSA), carries a higher risk of mortality. What is not clear is the cut-off BSA at which this risk becomes significant, or if the higher risk is related rather to larger confluent areas of epidermal necrosis rather than the total BSA. Another possibility is that the increased risk in TEN is associated with the more extensive mucosal erosions in TEN compared to SJS and SJS/TEN overlap. These need further evaluation as they may help clinicians looking after these patients to better prognosticate.

There is a suggestion that there may be an association between Gram-negative BSI and Gram-negative skin infection although this was not statistically significant. All 6 fatal cases were found to have Gram negative BSI, 2/6 grew a Gram negative organism from the skin. On the other hand there was no association between Gram-positive skin and blood cultures. These findings, although not conclusive, suggest that a Gram-negative skin culture is associated with a greater risk of BSI in SJS/TEN. This observation highlights the importance of including broad coverage of Gram negative micro-organisms in patients with suspected BSI to reduce associated mortality.

HIV infection was associated with SJS/TEN and mortality. HIV was more likely a a confounder as there is an increased risk of SJS/TEN in HIV-infected persons rather than direct impact of HIV on mortality. This study demonstrates the detrimental impact HIV and the associated opportunistic infections have had on the epidemiology of SJS/TEN. Within our population, the 3 most common offending agents were nevirapine, co-trimoxazole and anti-tuberculosis drugs; all drugs mainly used to treat AIDS and associated infections. Furthermore there were significantly more females who presented with SJS/TEN in this series. During the study period nevirapine-based antiretroviral therapy was the preferred regimen for women in the first trimester of pregnancy and those planning a pregnancy to prevent mother to child transmission of HIV infection. Our data shows that nevirapine was a common cause of SJS/TEN and this placed women at a disproportionately higher risk of developing SJS/TEN.

There are several limitations of our study, including those inherently associated with a retrospective study. A significant limitation of the study is that some parameters needed for SCORTEN, a validated severity of illness score for SJS/TEN were not available in this retrospective study, as a result the risk factors for mortality in our population could not be compared with other published studies [15]. Less than half of the patients who had skin cultures done had blood cultures done and vice versa introducing culture bias and resulting in inadequate sample size to allow for results reach statistical significance. Skin cultures were done at the discretion of the treating clinician on areas which looked infected and this may have introduced selection bias. The study population was at a single centre and all participants were treated by a small group of doctors, giving relative uniformity to case definitions, interpretation of laboratory results and management plans. The small sample size did not allow for an appropriate multivariable analysis to assess independent association of the variables associated with mortality.

## Conclusion

We describe characteristics and outcomes of SJS/TEN-associated skin cultures and BSI in a predominantly HIV-infected population in a low resource setting. We evaluated investigations and demographic factors inherently involved in predisposing patients to BSI and mortality. Percentage of denuded body surface area, Gram-negative blood cultures and active tuberculosis are risk factors of mortality within our population. There seems to be an association between Gram-negative BSI and Gram-negative skin infection but this needs larger studies. All these suggests that to manage these patients we need to address baseline co-morbidities as well as prevent and treat infections should they arise.
